# Immune reconstitution inflammatory syndrome associated with acquired immunodeficiency syndrome-related gastrointestinal limited Kaposi's sarcoma presenting as acute intestinal obstruction: a case report

**DOI:** 10.1186/1752-1947-5-327

**Published:** 2011-07-26

**Authors:** Jyotirmoy Pal, Ankit Shrivastav, Hari Shankar Pathak, Dipendra Kumar Sarkar

**Affiliations:** 1 Associate Professor, Department of Medicine, Institute of Postgraduate Medical Education & Research, Kolkata, West Bengal, India; 2 RMO cum Clinical Tutor, Department of Medicine, Institute of Postgraduate Medical Education & Research, Kolkata, India; 3 Associate Professor, Department of Medicine, KPC Medical College & Hospital, Kolkata, India; 4 Associate Professor, Department of Surgery, Institute of Postgraduate Medical Education & Research, Kolkata, India

## Abstract

**Introduction:**

Immune reconstitution inflammatory syndrome during anti-retroviral treatment of acquired immunodeficiency syndrome (AIDS) -associated gastrointestinal Kaposi's sarcoma has rarely been reported.

**Case Presentation:**

A 36-year-old Asian Indian male, newly diagnosed with AIDS and treatment naïve, was started on highly active antiretroviral therapy (HAART). He developed acute intestinal obstruction after four weeks of therapy. A laparotomy was done with excision and adhesiolysis leading to relief of symptoms. A histology report revealed the lesion to be Kaposi's sarcoma. Our patient was diagnosed to be having immune reconstitution inflammatory syndrome associated with AIDS-associated gastrointestinal limited Kaposi's sarcoma, which presented as acute intestinal obstruction. Our patient was treated with paclitaxel post-operatively and HAART was continued. Our patient responded to therapy.

**Conclusion:**

Immune reconstitution inflammatory syndrome involving Kaposi's sarcoma may occur in HAART-naïve individuals with AIDS-related Kaposi's sarcoma. Gastrointestinal Kaposi's sarcoma may present with sudden increase in size or inflammation leading to acute intestinal obstruction. This does not indicate failure of HAART or a need for changes in anti-retroviral regimen.

## Introduction

Kaposi's sarcoma (KS) remains the most common tumor in individuals infected with human immunodeficiency virus (HIV) and is associated with significant morbidity and mortality. Highly active anti-retroviral therapy (HAART) for acquired immunodeficiency syndrome (AIDS) decreases the incidence of KS, prolongs the time to treatment failure in KS, leads to resolution of individual lesions, and also decreases KS herpes virus viral load. Immune reconstitution inflammatory syndrome (IRIS) is well recognized as a complication of using HAART for treating AIDS, especially when associated with mycobacterial, fungal or viral infection. Our case demonstrates that KS can worsen during HAART-associated increases in the cluster of differentiation 4 (CD4) count and KS is an IRIS-associated disease. IRIS-KS may be associated with significant complications. It is important for clinicians to realize that KS-associated IRIS does not indicate failure of HAART or a need for changes in the anti-retroviral regimen. Instead, chemotherapy in conjunction with HAART can effectively control the symptoms of IRIS as well as resolve KS.

## Case presentation

A 36-year-old Asian Indian male patient attended our clinical immunology outpatient clinic with a fever of two months duration and two episode of herpes zoster in the last year. He also complained of significant weight loss in the last three months. He had no history of chronic cough or diarrhea. He was not a diabetic and not alcoholic. Our patient had a history of multiple unprotected sexual exposure around eight years ago.

On clinical examination his vitals were normal. He had a mild pallor and oral candidiasis. He had no significant lymphadenopathy or any cutaneous lesions. A systemic examination revealed no significant abnormality. Routine investigations were within normal limits. An ultrasound of his abdomen revealed no abnormality. An enzyme-linked immunosorbent assay (ELISA) for HIV-1 was positive and this was confirmed by western blot. Serology for Hepatitis B and C was non-reactive. Our patient's CD4 count was 67/mm^3^. He was put on HAART therapy consisting of nevirapine 30 mg twice a day, lamuvidine 150 mg twice a day and zidovudine 300 mg twice a day.

After 12 days our patient came back with a complaint of upper abdominal discomfort. An abdominal examination revealed mild epigastric tenderness, no organomegaly and normal peristaltic sound. An ultrasound of his abdomen was normal. He was presumed to be suffering from gastrointestinal side-effects, which is very common during initiation of HAART, and advised to take a proton pump inhibitor.

**Figure 1:**
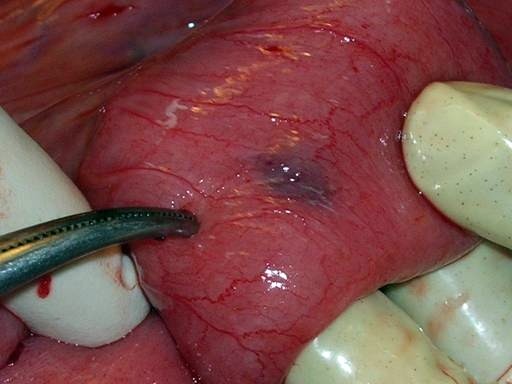
**Purple colored patches on the small bowel serosal surface seen during laparotomy**.

**Figure 2:**
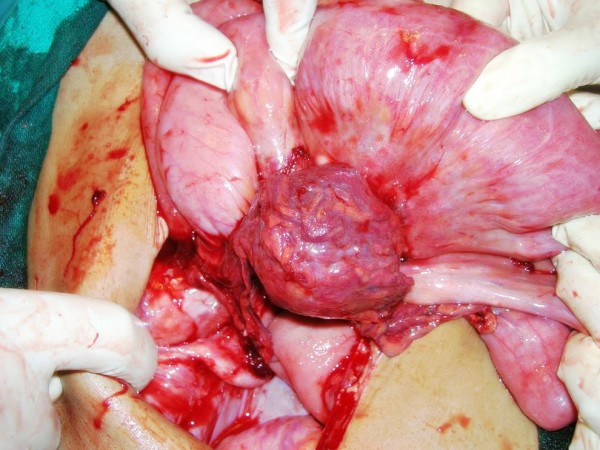
**Purple colored rounded cystic lesions lined by tortuous vessels seen on the small bowel mesentery during laparotomy**.

Our patient came back after another two weeks with severe abdominal pain, vomiting and abdominal distension. On examination his abdomen was found to be distended with absent peristaltic sound. An ultrasound of his abdomen was done and showed distended bowel loops with an admixture of air and fluid suggestive of acute intestinal obstruction. Routine investigations were normal and his CD4 count was 356/mm^3^. We put our patient on conservative management with fluids and nasogastric suction. However there was no improvement in 24 hours and our patient complained of increasing pain. A surgical consultation was taken and an exploratory laparotomy was performed with universal precautions. On exploration, small bowel loops were seen matted with a minimal amount of ascites. Purple colored patches, three in number, were seen on the serosal surface of his small bowel. Three thick walled, purple colored rounded cystic lesions adherent to surrounding bowel loops and omentum containing dirty white fluids were seen on the small bowel mesentery. The lesions had prominent veins over their surfaces (Figures 1 and 2). The lesions were excised; adhesiolysis was performed followed by peritoneal lavage with normal saline. The excised lesion was sent for histopathology.

**Figure 3:**
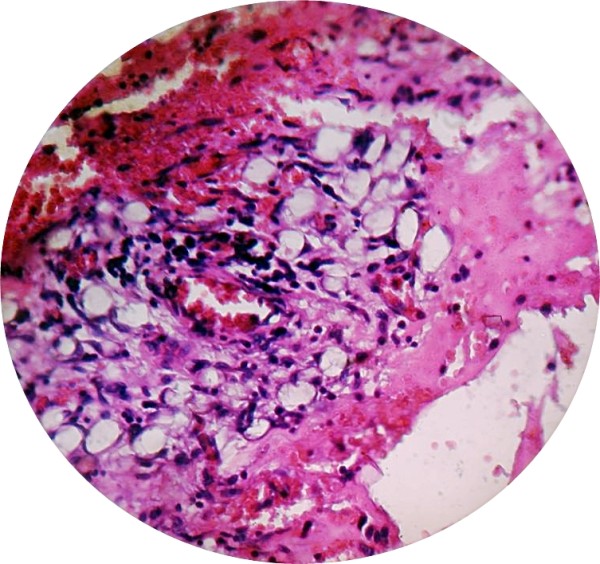
**Biopsy of excised lesion showing tortuous blood vessels, proliferation of pleomorphic endothelial cells with extra-vasation of red blood cells in stroma**. (H&E stain, 400×).

Our patient's symptoms improved after surgery. He was started on an oral diet after five days. The histological examination of the excised lesion showed tortuous blood vessels lined by a single layer of pleomorphic endothelial cells with extra-vasation of red blood cells in the stroma with spindle cells (Figure 3) suggestive of KS. His CD4 count was repeated and was 476/mm^3^. Our patient was started on paclitaxel (eight cycles of 100 mg/m^2 ^intravenously over three hours every two weeks) and the HAART was continued along with supportive management. He responded well to treatment and is doing well on follow-up.

## Discussion

KS is a multi-focal neoplastic disease that originates from the lymphatic endothelium, most frequently involving the skin. Also commonly involved are the mucous membrane, lymph nodes, gastrointestinal system and lungs. Lesions have been reported in virtually every organ including the heart and central nervous system. Human herpes virus-8 has been strongly implicated as a co-factor in the pathogenesis of KS. In HIV-infected patients, KS is an AIDS-defining illness.

Gastrointestinal involvement in KS is frequent in patients with advanced HIV disease. In one series of KS, gastrointestinal lesions were found in up to 51% of the cases [1]. Most clinical series have underestimated the overall incidence of luminal gastrointestinal involvement with KS because intestinal lesions rarely lead to symptoms. In a prospective endoscopic evaluation of 50 AIDS patients with KS, gastrointestinal tract involvement was present in almost all cases [2]. In another series, gastrointestinal involvement was reported in 40% of cases at initial diagnosis and up to 80% at autopsy.

Gastrointestinal involvement commonly occurs in association with cutaneous lesions or lymph node involvement, with gastrointestinal tract involvement alone occurring in only 3.5% of cases [3]. The absence of skin or lymph node KS, however, does not exclude the possibility of gastrointestinal involvement [2]. Gastrointestinal KS is mostly found in the stomach and duodenum with jejunum, ileum or large bowel rarely being involved. The biliary tract is also commonly involved. Lesions appear either as macule, sub-mucosal growth or as nodules [2]. Most of the lesions (80%) are clinically silent with bleeding, protein-losing enteropathy, mal-absorption and obstructive jaundice being the most common presentation. Gastrointestinal obstruction has also been rarely reported [2].

IRIS is an inflammatory reaction to an opportunistic pathogen and/or tumor antigen that occurs early after initiation of HAART in patients with AIDS and is temporally related to an increase in the host's CD4+ lymphocyte count [4]. IRIS is most frequently observed in individuals with severe CD4+ T-cell depletion and is believed to be due to reconstitution of immune responses to a previously existing (but clinically occult or previously treated) pathogen or tumor antigen, rather than development of a new opportunistic infection or progression of opportunistic infection due to treatment failure. Our patient had AIDS and started on HAART therapy. He was admitted with severe pain abdomen after four weeks of HAART and diagnosed to be having intestinal obstruction. An emergency laparotomy was done which showed matted small bowel loops with purple colored patches and cysts with adhesions on the small bowel serosal surface and mesentery. Adhesiolysis and resection were done and the lesion sent for histopathological examination, which showed it to be a case of KS. Our patient had no features of KS during initiation of HAART. An ultrasound of his abdomen was also normal.

Initiation of HAART is usually associated with a regression of KS. However in this case there was probably a rapid increase in the size of the KS lesion, causing intestinal obstruction. Several features of this case suggest that the worsening symptoms and clinical finding represented IRIS rather than progressive KS. The rise in his CD4 count and the temporal relationship of bowel obstruction to HAART initiation also support the diagnosis of KS-IRIS.

Although KS is prevalent among HIV-1 infected persons, IRIS during anti-retroviral treatment of AIDS-associated KS has only been reported three times [5-7]. In one case, laryngeal obstruction occurred in a patient with known KS shortly after initiation of HAART [5]. In the second case, parotid gland KS developed in an individual two years after initiation of HAART, despite there being good CD4+ lymphocyte reconstitution and virus suppression [6]. In the third case, rapidly progressive KS lesions with lymphadenopathy and tissue swelling occurred in a patient during anti-retroviral treatment, despite an increased CD4+ lymphocyte count and decreased HIV-1 level and KS-associated herpes virus replication [8].

In a review of 5,832 patients with AIDS undergoing HAART, Bower *et al. *identified 150 therapy-naïve patients with a first presentation of KS and recorded their clinic-pathologic features prospectively[9]. They identified ten patients with IRIS-KS in the patient cohort of HIV patients with KS who were started on HAART.

This is a rare case of IRIS associated with AIDS-related gastrointestinal limited KS, presenting as an acute intestinal obstruction. It is likely that KS-associated IRIS is more common than the literature reflects due to limited awareness of this condition. It is important for clinicians to realize that KS-associated IRIS does not indicate failure of HAART or a need for changes in the anti-retroviral regimen. Instead, chemotherapy in conjunction with HAART can effectively control the symptoms of IRIS as well as resolve KS, especially when KS-IRIS is severe or there is visceral involvement.

## Conclusions

IRIS-KS may occur in HAAR- naïve individuals with AIDS-related KS. Gastrointestinal KS may present with a sudden increase in size leading to acute intestinal obstruction. KS-associated IRIS does not indicate failure of HAART or a need for changes in the anti-retroviral regimen.

## Consent

Written informed consent was obtained from the patient for publication of this case report and any accompanying images. A copy of the written consent is available for review by the Editor-in-Chief of this journal

## Competing interests

The authors declare that they have no competing interests.

## Authors' contributions

JP and AS were involved in patient care and management. They also conceived the report and were involved in preparation of the manuscript. DKS was involved in the surgery and patient management. HSK helped diagnose our patient and review the manuscript. DG was involved in counseling and administration. All authors read and reviewed the manuscript.
